# Cocci: Their Demonstration and Significance

**Published:** 1907-07-06

**Authors:** 


### Pathology in General Practice.

### COCCI: THEIR DEMONSTRATION AND SIGNIFICANCE. 

(*Concluded from p.* 261.)

The gonococcus is in many ways one of the most important of the specific cocci, both the various acute troubles it gives rise to, and the late results which may follow these, often being of the utmost importance to the practitioner. Gonorrhœa is a much more serious complaint than is generally supposed, and it may often be a question of the greatest difficulty to say whether or not, especially in the chronic cases, the source of infection has disappeared and the person is safe. The neglect of careful examination and advice in such examples may often lead to dire mischief to a young wife, the results of which are too often permanent and the starting-point of chronic ill-health for the rest of life. The question of sterility, the medico-legal bearing of cases of criminal rape on infants by people suffering from gonorrhœa, the question of gonorrhœal conjunctivitis are a few of the other points which may arise and require investigation at any time; and all this being so, it will be readily understood how important it is to be able to detect this organism.

### The Gonococcus.

The gonococcus is a small organism, usually occurring in diplococcic form, the two opposed sides being flattened or slightly concave, giving the appearance of two little beans or two little kidneys placed side by side. Both tetrads and single specimens may be seen, but they are much rarer than the typical picture just described. Gonococci stain readily with any of the simple stains, perhaps the best of these being carbol thionin, and they lose—*i.e.*, do not stain—by Gram's stain; this latter peculiarity is a diagnostic point of great value and one which should never be omitted in a routine examination. Another differential stain recommended by Schütz is as follows:—(1) Sat. aq. methylene blue in 5 per cent. carbolic. Five to ten minutes. (2) Wash. (3) Decolorise with acetic acid, 5 drops in 20c.c. water. Three seconds. (4) Wash, dry and mount.

The gonococci by this method are blue, other organisms unstained. The organisms are demonstrated in the usual manner by making smears of the suspected discharge; they are present in large numbers in the acute cases, male or female, and have this peculiarity—namely, that they are almost all contained within the polymorphonuclear leucocytes; that is, in the cells that form the pus. In the female it is of little use taking an ordinary swab from the vagina; the sites to be selected are the urethra and cervix, and the practitioner must remember this in order to avoid confusion or a wrong diagnosis. A case in point illustrative of this was that of a girl (unmarried) with a discharge from the vagina. In the first instance an ordinary vaginal smear was negative as regards the gonococcus, but a subsequent one from the urethra showed absolutely typical and very numerous gonococci; hence if a diagnosis had been given on the first specimen alone, it would have been a very fallacious one. After the acute symptoms have passed off, the purulent discharge gradually disappears and is replaced by a thin glairy one, which in turn may quickly disappear or pass on to a definite gleet, or only a "morning drop."

### Difficulties.

Here the difficulty often begins; in some instances typical gonococci may be readily detected in such discharges, but in others, owing to the addition of other cocci or bacilli (mixed infections), it may not be so easy, and one's differential stains must then be used to determine the point. Further, by now, as the case has become chronic (the morning drop, for example), the microscopic character of the secretion has altered, and many large squamous cells have taken the place of the previous abundant pus-cells, this desquamation being due to the passage of the cocci down through the mucous membrane into the deeper layers, where they have set up inflammatory changes. In such a case the gonococci may be seen lying on the dead squamous cells, or free, or a few may still be in the pus-cells, or none may be detected at all. Though absent, it is not safe to say the person is cured; in many instances of this kind a night's indulgence in alcohol or sexual intercourse will bring back the purulent discharge almost at once, and typical gonococci may again be detected. The explanation of this is simply that the organisms have not really been absent, but have been lying hidden in the crypts of the glands, in the deeper layers of the mucosa, or in the prostate, where any excitant may quickly stir them into activity again, and so cause a renewal of the symptoms. Some of the very chronic cases are very hopeless, the infection persisting and persisting (Wertheim cultivated them from a case of two years' standing), and here other cocci are generally present, aggravating and keeping up the condition. It has recently been shown that the gonococcus may have a much wider distribution in man than was formerly supposed. Leaving out of account conjunctivitis cases, which are caused by direct infection and which may be easily diagnosed by suitably stained smears, the organism has been found in joints, sheaths of tendons, ulcerative endocarditis, pus from the pleura, peritonitis (females) from spread through the Fallopian tubes which may also contain pus (pyosalpinx), abscesses in epididymis, periurethral abscesses, etc.

The last coccus we may mention is the Diplococcus Intracellularis Meningitidis of Weichselbaum, the usually accepted cause of epidemic cerebro-spinal meningitis. Morphologically it resembles the gonococcus somewhat closely, being like it in shape, also being found in the pus-cells, and by not staining by Gram's stain. It may be demonstrated in smears taken from the purulent material drawn off by lumbar puncture of such cases, thionic blue, or any of the other common stains showing it up quite clearly.

